# Tailored Therapy vs. Empirical Therapy for *Helicobacter pylori* Eradication: An Umbrella Review of Systematic Reviews and Meta-Analyses

**DOI:** 10.3390/jpm15100458

**Published:** 2025-09-30

**Authors:** Dmitrii N. Andreev, Alsu R. Khurmatullina, Igor V. Maev, Dmitry S. Bordin, Andrey V. Zaborovsky, Yury A. Kucheryavyy, Philipp S. Sokolov, Petr A. Beliy

**Affiliations:** 1 Department of Internal Disease Propaedeutics and Gastroenterology, Russian University of Medicine, 127473 Moscow, Russia; 2 Department of Pancreatic, Biliary and Upper Digestive Tract Disorders, A. S. Loginov Moscow Clinical Scientific Center, 111123 Moscow, Russia; 3 Department of General Medical Practice and Family Medicine, Tver State Medical University, 170100 Tver, Russia; 4 Department of Pharmacology, Russian University of Medicine, 127473 Moscow, Russia; 5 Ilyinskaya Hospital, 143421 Krasnogorsk, Russia

**Keywords:** *Helicobacter pylori*, tailored therapy, empirical therapy, susceptibility-guided therapy, eradication rate, umbrella review

## Abstract

**Objective:** This study aimed to provide the first umbrella review to systematically evaluate the evidence for the efficacy of personalized susceptibility-guided tailored therapy (TT) versus empirical therapy (ET) for *Helicobacter pylori* eradication. **Methods:** An umbrella review was conducted following Joanna Briggs Institute standards, registered in PROSPERO (CRD420251104335). A comprehensive literature search across MEDLINE/PubMed, EMBASE, RSCI, and Google Scholar identified systematic reviews and meta-analyses published between 1 January 1985 and 10 June 2025. Studies comparing TT and ET were included. Methodological quality was assessed using a modified AMSTAR-2 tool, GRADE, and the risk of bias was evaluated using the ROBIS tool. Data were synthesized using a random-effects model, with heterogeneity assessed using the I^2^ statistic. **Results:** A total of 7 systematic reviews and meta-analyses were included, covering 66 primary studies. TT was associated with a significantly higher eradication rate compared to ET (RR = 1.265; 95% CI: 1.137–1.407). In first-line treatment, TT showed consistent superiority (RR = 1.156; 95% CI: 1.117–1.196), which was further supported by high-quality meta-analyses (RR = 1.288; 95% CI: 1.022–1.624). The benefit in second-line therapy did not reach statistical significance (RR = 1.291; 95% CI: 0.834–1.999). The absolute eradication rates were 84.31% (95% CI: 80.94–87.41) for TT and 67.80% (95% CI: 58.48–76.46) for ET. **Conclusions:** TT is more effective than ET in the first-line *H. pylori* eradication regimen. However, the benefit is less evident in second-line regimens.

## 1. Introduction

*Helicobacter pylori* (*H. pylori*) is a globally prevalent bacterial pathogen responsible for chronic gastritis and peptic ulcer disease, and is a well-established risk factor for gastric adenocarcinoma [1,2]. Despite advances in sanitation and eradication efforts, infections remain highly endemic, with meta-analyses estimating that approximately 40% of adults [3] and 30–35% of children and adolescents [4] worldwide are infected with *H. pylori*. Eradication of *H. pylori* has been shown in randomized trials and pooled analyses to reduce the incidence of gastric cancer by roughly 40–50% over long-term follow-up (relative risk for gastric cancer: 0.61%) (95% CI: 0.47–0.79) [5], underscoring the relevance of effective treatment strategies.

However, a significant rise in antibiotic resistance among *H. pylori* strains poses a major challenge to empirical therapy (ET). A comprehensive systematic review and meta-analysis of 163 studies, including 47,002 isolates from 36 countries, reported pooled resistance rates of 26.7% (95% CI 24.7–28.8) for clarithromycin, 59.6% (55.2–63.9) for metronidazole, and 26.2% (23.5–28.9) for levofloxacin [6]. Over the past decade, these resistance rates continue to increase, leading to a further decline in the effectiveness of empirical treatment regimens [7,8].

In response, both the Maastricht VI/Florence Consensus Report (2022) guidelines and the ACG guidelines (2024) advocate for susceptibility-guided, tailored therapy (TT), whereby treatment is informed by the culture-based or molecular detection of resistance mutations in gastric biopsy specimens (e.g., clarithromycin 23S rRNA; gyrA for fluoroquinolones) [2,9]. Initial studies dating from the 1990s, by Graham et al. (1992) [10] and Glupczynski et al. (1991) [11], demonstrated improved eradication rates with TT versus ET in first-line settings. In later studies, multiple meta-analyses have evaluated TT against ET, with most showing a clear benefit of TT in first-line regimens (relative risk of eradication 1.13; 95% CI 1.08–1.17) [12] but more mixed results in second- or third-line settings [13].

The present umbrella review aims to synthesize existing meta-analyses comparing tailored therapy and empirical therapy for *H. pylori* eradication and to quantify their relative efficacy across different treatment lines.

## 2. Materials and Methods

### 2.1. Search Strategy

We conducted an umbrella review in accordance with Joanna Briggs Institute standards [14]. This design is chosen when several systematic reviews address closely related questions; it collates their results, highlights consistencies or discrepancies, and pinpoints remaining knowledge gaps, thereby guiding clinicians, policymakers, and investigators. We applied current best-practice methods, consistent with established umbrella reviews [15]. The umbrella review was registered in advance in the PROSPERO database under the identification number CRD420251104335. We carried out an extensive literature search in accordance with the PRISMA 2020 guidelines for systematic reviews and meta-analyses (File S1) [16]. A completed PRISMA-P checklist is available in the Supplementary Materials, Table S1.

To ensure comprehensive coverage of the relevant literature, we searched across multiple databases, including MEDLINE/PubMed, EMBASE, the Russian Science Citation Index (RSCI), and Google Scholar. The search was restricted to studies published between 1 January 1985 and 10 June 2025, in order to focus on up-to-date evidence while maintaining a practical scope.

The next search strategy was conducted using MEDLINE/PubMed: (“Helicobacter pylori”[Mesh] OR “H. pylori” OR Helicobacter) AND (“tailored therapy” OR “personalized therapy” OR “susceptibility-guided therapy” OR “culture-guided therapy”) AND (“empirical therapy” OR “standard therapy” OR “eradication therapy”) AND (“meta-analysis”[Publication Type] OR “systematic review” OR “review”). Similar strategies were conducted when using other databases.

### 2.2. Eligibility Criteria and Quality Assessment

The methodology was based on the PICO approach. The population included individuals with or without a diagnosed clinical condition, provided that the systematic reviews explicitly stated their analyses were conducted on human subjects; studies involving animals were excluded. The intervention focused on any form of TT for *Helicobacter pylori* eradication. The comparison group included studies assessing standard or ET regimens. The outcomes of interest were eradication rates, typically expressed as odds ratios or relative risk, along with measures of heterogeneity and study quality. The study design was restricted to systematic reviews, with or without meta-analyses, which included randomized and non-randomized controlled clinical trials. Observational studies and other non-experimental designs were excluded. No language restrictions were applied.

Studies were excluded if they did not meet the methodological standards of systematic reviews or if they lacked a clear comparison between TT and ET regimens. Reviews that included only observational, retrospective, or case report designs were not considered. Studies that focused solely on in vitro or animal research, without human clinical data, were also excluded. Additionally, reviews with insufficient methodological transparency, unclear outcome reporting, or those that failed to specify the population or intervention strategy were excluded.

Two independent reviewers (F.S.S. and P.A.B.) assessed the methodological quality of the included systematic reviews and meta-analyses using a modified version of the AMSTAR-2 tool (A Measurement Tool to Assess Systematic Reviews) [17]. This instrument consists of 16 items, each rated as “yes” for full adherence, “no” if the criterion was unmet or insufficient data were provided, and “partial yes” when only some elements of the standard were fulfilled. To evaluate the interrater reliability before reaching consensus, we calculated both the kappa statistic (κ) and percentage agreement. A κ value above 0.7 was interpreted as strong agreement, between 0.5 and 0.7 as moderate, and below 0.5 as low reliability.

### 2.3. Risk of Bias Evaluation

The risk of bias for the included randomized controlled trials was assessed using the RoB 2.0 tool (Risk of Bias 2.0 for randomized trials) [18]. This tool evaluates five domains: (1) bias arising from the randomization process, (2) bias due to deviations from intended interventions, (3) bias due to missing outcome data, (4) bias in measurement of the outcome, and (5) bias in selection of the reported result. Each domain includes structured signaling questions to guide judgments, which are categorized as “low risk,” “some concerns,” or “high risk” of bias. Certainty of evidence was assessed for all associations with the updated Grading of Recommendations Assessment, Development, and Evaluation (GRADE) tool [19].

Two independent reviewers (P.S.S. and A.Z.V.) conducted the assessments. Disagreements were resolved by discussion and consensus.

To present the risk-of-bias assessments, we utilized the ROBIS tool, which enables clear visualization of domain-level and overall bias judgments across studies through traffic light plots.

### 2.4. Overlap of Primary Studies

The overlap of primary studies included in the systematic reviews was assessed using the Graphical Representation of Overlap for OVErviews (GROOVE) tool [20]. Based on an evidence matrix, GROOVE identifies the total number of primary studies and systematic reviews, the absolute number of overlapping and non-overlapping studies, and calculates the corrected covered area (CCA) to quantify the degree of overlap.

In our analysis, the CCA was calculated to be 19.95%, which corresponds to a very high degree of overlap. This level of overlap suggests that the findings across the included systematic reviews are highly reproducible and reliable, as multiple reviews consistently include and report on the same primary studies.

### 2.5. Data Extraction

Two independent reviewers (A.R.K. and D.N.A.) conducted the initial screening process to determine the relevance of systematic reviews and meta-analyses. This first phase involved evaluating titles, abstracts, and keywords. In cases of disagreement or when abstracts lacked sufficient detail, the full article was reviewed. A second round of screening focused on full-text analysis to verify that studies met the inclusion criteria. Data extraction was performed following a standardized protocol to capture the most relevant information from each study.

From each included study, we extracted the publication year, number of included studies, TT methodology, drug selection method for TT, and comparative effectiveness of ET as relative risk (RR) with 95% confidence intervals. We also collected data on the total population size, the number of participants in the TT and ET groups, the number of effective cases in each group, and the meta-analysis model used. To avoid duplication, results were examined for repetition across multiple reviews or meta-analyses. When identical effect sizes appeared in more than one source, only one was retained. If differing effect sizes were found, data from the original primary study were used.

Due to the considerable variation in study designs, participant characteristics, and outcome measures across the included studies, a random-effects model was employed for data synthesis. Heterogeneity among studies was evaluated using the Q statistic, I^2^ coefficient, and prediction intervals.

Publication bias was assessed using several complementary approaches, including funnel plots and the Egger test. Confidence intervals (95% CIs) were calculated for all effect size estimates, and the threshold for statistical significance was set at *p* < 0.05. All statistical analyses were conducted using MedCalc software (version 23.2.6; Ostend, Belgium).

## 3. Results

### 3.1. Study Selection

**Figure 1:**
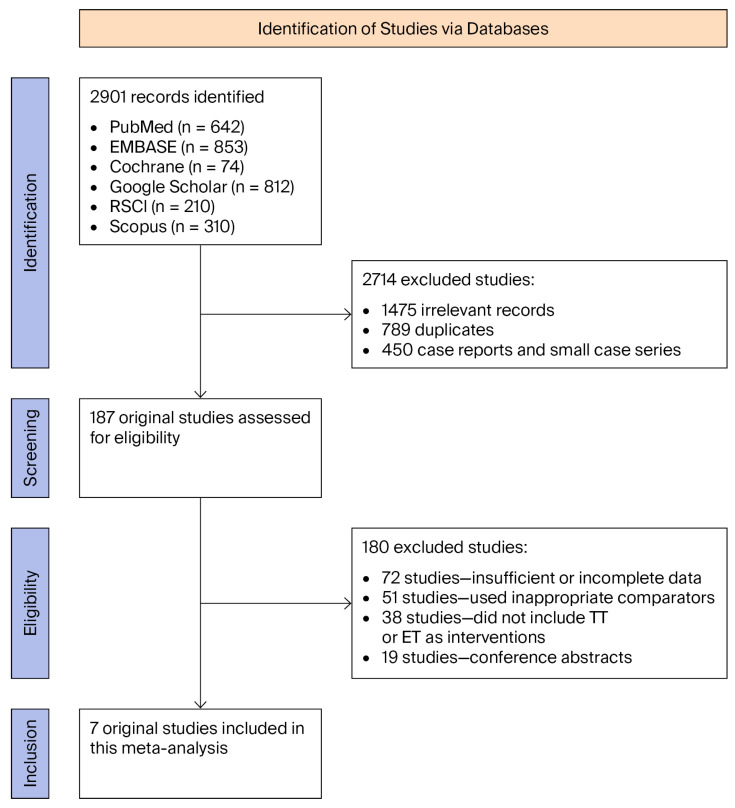
PRISMA (Preferred Reporting Items for Systematic Reviews and Meta-Analyses) flowchart.

The initial literature search revealed 2901 records, including 642 from PubMed, 853 from EMBASE, 74 from the Cochrane Library, 812 from Google Scholar, 210 from RSCI, and 310 from Scopus. After removal of duplicates and initial screening of titles and abstracts, 2714 records were excluded. Among these, 1475 were considered irrelevant, including 512 that did not investigate the target patient population, 426 that did not include *H. pylori* eradication, 318 that did not report relevant clinical outcomes, and 219 that were non-original articles such as reviews, editorials, or conference abstracts. Additionally, 789 duplicates and 450 case reports or small case series were excluded. Full-text assessment of the remaining articles led to the exclusion of 180 studies, comprising 72 with insufficient or incomplete data, 51 using inappropriate comparators, 38 that did not include TT or ET as interventions, and 19 conference abstracts. Following this rigorous selection process, a total of seven studies were included in both the qualitative synthesis and the quantitative meta-analysis (Figure 1).

Table 1 summarizes the key characteristics of the included systematic reviews and meta-analyses, including study type, design, sample size, intervention (ET and TT), risk of bias assessments, and quality evaluations.

### 3.2. Robis Assessment

**Figure 2:**
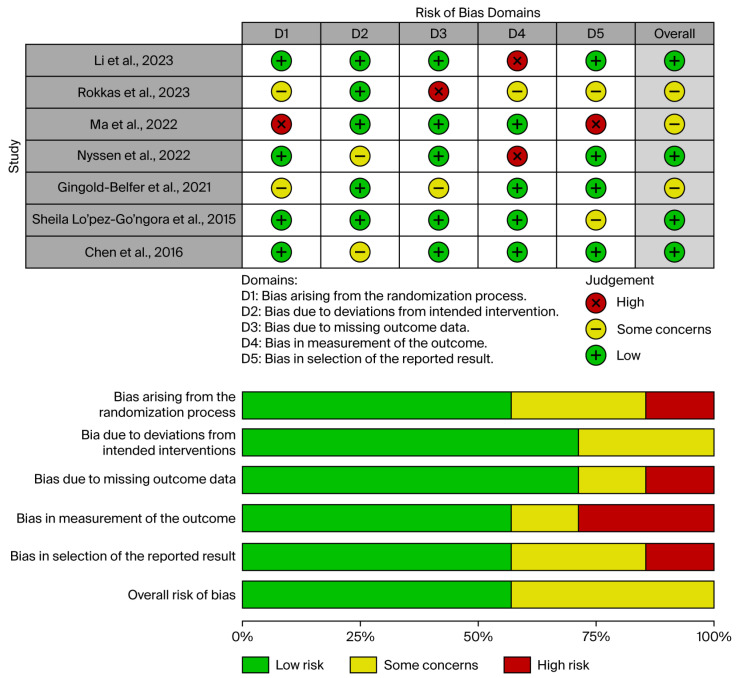
Overview of the bias risk assessment conducted using the ROBIS tool [12,13,21,22,23,24,25].

Figure 2 provides an overview of the bias risk assessment conducted using the ROBIS tool. Among the seven studies reviewed, four (57%) were rated as having a low risk of bias, and two (43%) had an unclear risk. The greatest concerns regarding bias were found in the domains of “Measurement of Outcome”. In contrast, the domain addressing “Bias due to Deviations from Intended Interventions” demonstrated the lowest level of bias. Interrater agreement in assessing bias was strong, with a kappa coefficient of 0.92. The ROBIS assessment is presented in Figure 2.

### 3.3. Groove Analysis

**Figure 3:**
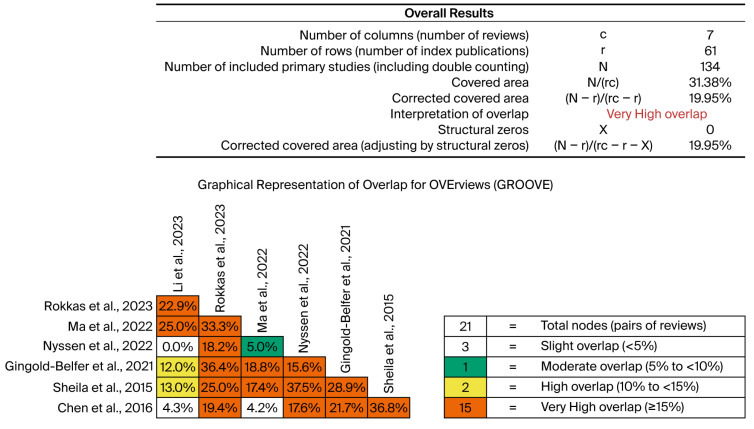
Graphical visualization of the GROOVE findings [12,13,21,22,23,24,25].

A total of 66 primary studies were identified across the included systematic reviews and meta-analyses, of which 28 were unique. The overall degree of overlap within the evidence matrix was very high, with a corrected covered area (CCA) of 19.95%. After accounting for chronological structural missingness, the adjusted CCA remained within the same range. A graphical visualization of the GROOVE findings is presented in Figure 3.

### 3.4. Effectiveness of TT Compared to ET

**Figure 4:**
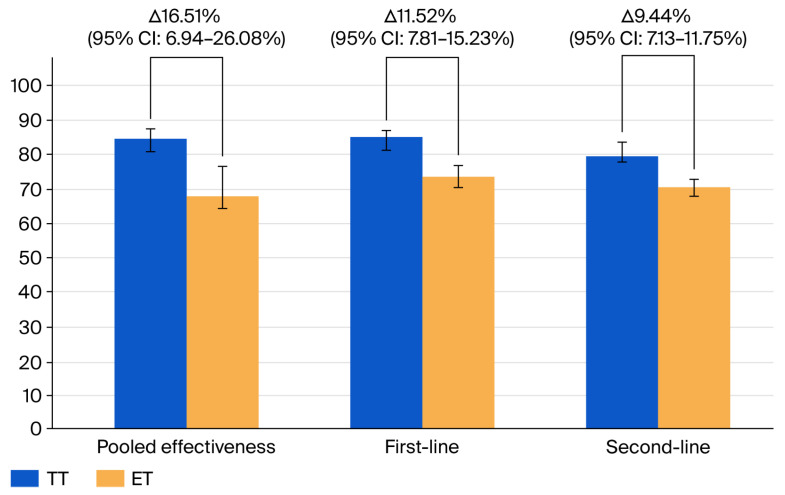
Proportional comparison of TT vs. ET outcomes.

The overall pooled effectiveness of TT compared to ET demonstrated a statistically significant benefit in favor of TT, with a relative risk (RR) of 1.265 (95% CI: 1.137–1.407); the heterogeneity level was as high as 97.68% (S2). Figure 4 presents a comparison of effectiveness between the generalized first- and second-line treatments, shown as graphs illustrating the corresponding proportions.

Importantly, when restricting the analysis to high-quality meta-analyses only (including three studies), the benefit of TT over ET was assessed, with a pooled RR of 1.288 (95% CI: 1.022–1.624).

Across all comparisons, heterogeneity was substantial, with I^2^ values exceeding 90%, indicating considerable variability among the included studies.

The funnel plot showed no clear evidence of publication bias. Although there was some asymmetry, this may reflect underlying heterogeneity in study outcomes rather than bias. A substantial portion of the effect sizes were non-significant. The absence of publication bias was further supported by the results of Egger’s test (*p* = 0.1663).

All data presented here are available as graphical representations in the Supplementary Materials.

To further evaluate the effectiveness of treatment strategies for *H. pylori* eradication, we additionally compared the pooled prevalence of successful eradication achieved with TT versus ET (Table 2). Across all included studies (*n* = 7 meta-analyses), the pooled eradication rate for TT was 84.31% (95% CI: 80.94–87.41), compared to 67.80% (95% CI: 58.48–76.46) for ET. RR of successful eradication with TT was 1.27 (95% CI: 1.14–1.41), with a highly significant *p*-value (*p* < 0.0001). However, heterogeneity was substantial (I^2^ = 99%, 95% CI: 98.66–99.25).

When stratified by treatment line, TT remained superior to ET in first-line regimens (n = 4), with a pooled RR of 1.156 (95% CI: 1.117–1.196) and eradication rates of 85.09% (95% CI: 82.81–87.23) for TT and 73.57% (95% CI: 70.03–76.96) for ET. Although TT showed higher eradication rates in the second-line setting 79.75%, 95% CI: 77.38–82.02) compared to ET, the effectiveness was not statistically significant, yielding a pooled RR of 1.291 (95% CI: 0.834–1.999).

Among high-quality meta-analyses (n = 3), TT also demonstrated better performance, with an RR of 1.29 (95% CI: 1.02–1.62; *p* < 0.0001). The eradication rate in the TT group was 84.14% (95% CI: 79.79–88.06) compared to 66.85% (95% CI: 50.33–81.47) for ET.

### 3.5. Sensitivity Analysis

To address concerns regarding potential study overlap and the associated risk of double-counting, we conducted a sensitivity analysis in which overlapping primary trials were systematically excluded before re-estimating the pooled effects.

After removing overlapping studies, the pooled effectiveness estimates were as follows: for first-line therapy, TT achieved a total effective rate of 81.82% (95% CI: 77.08–86.12) compared with 73.22% (95% CI: 71.82–74.61) for ET; for second-line therapy, TT showed 79.49% (95% CI: 76.59–82.25) versus 70.97% (95% CI: 59.29–81.38) for ET.

When compared with the original pooled analyses that included overlapping studies—first-line TT: 85.09% (95% CI: 82.81–87.23), ET: 73.57% (95% CI: 70.03–76.96); second-line TT: 79.75% (95% CI: 77.38–82.02), ET: 70.31% (95% CI: 62.03–77.97)—the overlap-adjusted results showed a slight reduction in efficacy for first-line therapy, with TT decreasing from 85.09% to 81.82%. In contrast, second-line estimates remained almost unchanged (TT: 79.49% vs. 79.75%; ET: 70.96% vs. 70.31%). Overall, these findings suggest that study overlaps had minimal impact on the results.

Thus, exclusion of overlapping studies confirms the robustness of the observed benefit of TT over ET.

## 4. Discussion

To our knowledge, this is the most comprehensive umbrella review summarizing and evaluating the certainty of evidence for TT and ET effectiveness in *H. pylori* eradication, where TT demonstrated consistently higher eradication rates in first-line treatment, particularly when based on genotypic resistance profiling.

Li et al. reported significantly higher eradication rates with TT when triple therapy was applied (*p* < 0.0001; RR = 1.20; 95% CI: 1.12–1.29). By contrast, when quadruple therapy was used, ET regimens yielded superior outcomes (*p* = 0.001; RR = 0.93; 95% CI: 0.89–0.97), regardless of bismuth inclusion. No significant differences were observed in the overall rate of adverse events (34% vs. 37%; *p* = 0.17).

Ma et al., analyzing more than six thousand patients, reported eradication rates of 86% in the susceptibility-guided group compared to 76% in the ET group (RR = 1.14; 95% CI: 1.08–1.21). However, when comparing susceptibility-guided therapy to contemporary ET regimens (both with and without bismuth), the difference was no longer statistically significant (RR = 1.05; 95% CI: 0.84–1.30).

Nyssen et al. further supported these findings, showing that while TT was generally superior to ET (eradication rate: 86% vs. 76%; RR: 1.12; 95% CI: 1.08–1.17), its relative benefit was attenuated when compared specifically to optimized empirical quadruple therapy excluding suboptimal triple regimens (RR: 1.04; 95% CI: 0.99–1.09), with similar results seen in RCT-only analyses (RR: 1.05; 95% CI: 0.99–1.12). In rescue settings, the two strategies performed comparably (RR: 1.09; 95% CI: 0.97–1.22).

In a broader analysis by Gingold-Belfer et al., TT outperformed 7–10-day triple ET (RR = 1.14; 95% CI: 1.07–1.21), though evidence for its superiority in second-line settings was inconclusive due to heterogeneity (I^2^ = 87%).

The optimal standard for eradication therapy is an efficacy rate exceeding 90%, which has been recognized as the clinical standard in international guidelines [26,27]. However, even with TT, this threshold is not consistently achieved, which may reflect the influence of additional factors, such as treatment duration and PPI dosage [28]. Patient-specific factors influence treatment success. Among them, patient adherence is paramount: according to the Hp-EuReg registry, adequate compliance (≥90% of doses) was identified as the strongest independent predictor of eradication success, with more than a sixfold increase in the odds of cure compared to poor adherence (OR = 6.3; 95% CI: 5.2–7.7) [29,30]. In addition, common comorbidities such as type 2 diabetes mellitus are highly prevalent conditions worldwide, and obesity has consistently been associated with reduced eradication efficacy [31,32]. Because these patient-specific factors cannot be fully excluded or controlled, comparative studies of TT versus ET are inevitably exposed to residual confounding, which may bias the observed treatment effects.

The Maastricht VI/Florence Consensus Report recommends TT in regions with high resistance or after treatment failure, but notes barriers: cost, infrastructure, sampling errors, discordance between gastric and stool isolates, and the fact that resistance is not the sole determinant of success (drug pharmacokinetics, patient compliance, and bacterial load) [2]. Although TT represents a rational, potentially superior approach in first-line therapy [33], particularly in areas with known high resistance, its practical advantages must be weighed against logistical and economic constraints [34]. In well-resourced settings, TT may become standard; in others, optimized ET, guided by regional surveillance data, remains a cornerstone of *H. pylori* eradication strategy [35].

ET, especially when based on local resistance data and employing bismuth-containing quadruple regimens or concomitant therapies, remains a practical and often effective option [36,37,38]. Its strengths lie in accessibility, cost-effectiveness, and ease of implementation [39]. However, its major limitation is the potential for antibiotic misuse, which can further drive resistance, especially when outdated regimens (such as clarithromycin-based triple therapy in high-resistance areas) are still being used [40].

While susceptibility-guided therapy generally demonstrates higher eradication rates, Rokkas et al. emphasized that rates above 90% were achieved in fewer than half of the studies, and rates above 95% were reached in only a small minority. This highlights a critical limitation: TT, despite its potential, does not consistently achieve cure rates exceeding recommended thresholds unless optimized in accordance with antimicrobial resistance patterns and drug pharmacodynamics [41].

Future research should prioritize head-to-head trials comparing contemporary optimized regimens and explore cost-effectiveness models to better guide clinical decision making.

We present a first comprehensive analysis of the effectiveness of TT versus ET for *H. pylori* eradication, synthesizing current evidence from multiple comparative studies. The use of standardized methods to assess study quality, along with statistical tools to evaluate heterogeneity and risk of bias, enhances the reliability of our findings. Nevertheless, several limitations exist. Although TT generally demonstrates superiority over ET, achieving the clinically optimal standard of >90% remains challenging, particularly in the setting of insufficient treatment duration, suboptimal PPI dosing, or poor adherence. The quality of included studies varied, and high heterogeneity in treatment regimens, populations, and resistance detection methods may limit the generalizability of the results. Additionally, differences in the assessment of eradication, using various diagnostic methods, may have influenced the reported effect sizes.

## 5. Conclusions

In line with our findings, the current evidence supports the use of TT (particularly in first-line treatment and settings with known antibiotic resistance). However, when optimal ET regimens are selected, the added benefit of TT may be minimal. These results emphasize the need for individualized therapeutic approaches that incorporate local resistance data, prior treatment history, and pharmacogenetic considerations to enhance eradication success.

## Figures and Tables

**Table 1:** Characteristics of the systematic reviews.

Study, Year	Total Number of Included Studies	Methodology for TT	ET Type	Total Population, *n*	TT Total, *n*	TT Effective, *n*	ET Total, *n*	ET Effective, *n*	Heterogeneity, I^2^	Types of Included Studies	Quality Evaluation, AMSTAR-2	Quality Evaluation, GRADE
Li et al., 2023 [21]	12	Molecular methods	First and second line	3940	1780	1414	2160	1599	84%	RCT	High	High
Rokkas et al., 2023 [22]	34	Culture + PCR (gastric biopsy)	First and second line	9613	4875	3770	4738	2419	83.87%	RCT + non-RCT	Moderate	Low
Ma et al., 2022 [23]	21	18: cultural; 3: molecular	First line;	6005	2948	2462	2277	1701	72.2%	RCT	Low	Low
			second line		436	345	435	333	80.6%			
Nyssen et al., 2022 [12]	54	36: cultural; 16: molecular	First line	14,600	6705	5771	7895	6006	83%	27 RCT; 9 non-RCT	High	High
			Second line						78%			
Gingold-Belfer et al., 2021 [13]	16	14: cultural; 2: genetic	First line	4825	2374	2009	2451	1814	75%	11 RCT; 5 non-RCT	Moderate	Moderate
			Second line		342	274	468	305				
Sheila Lo’pez-Go’ngora et al., 2015 [24]	12	n/s	First line	1958	860	767	1098	849	33%	10 RCT; 3 quasi-RCT	Moderate	High
			Second line	455	207	169	248	150	87%			
Chen et al., 2016 [25]	13	10: cultural; 3: genetic	First line	3512	1085	904	1930	1317	57%	10 RCT; 3 non-RCT	High	Moderate
			Second line		161	127	300	234				

**Table 2:** Comparison of included studies.

Type of Comparison, n of Included Meta-Analyses	RR (95% CI)	Pooled TT Efficiency in Percentages and 95% CI	Pooled ET Efficiency in Percentages and 95% CI	Heterogeneity for RR	*p*-Value for RR
All studies, seven	1.27 (95% CI: 1.14–1.41)	84.31% (95% CI: 80.94–87.41)	67.80% (95% CI: 58.48–76.46)	99% (95% CI: 98.66–99.25)	*p* < 0.0001
First-line, four	1.16 (95% CI: 1.12–1.20)	85.09% (95% CI: 82.81–87.23)	73.57% (95% CI: 70.03–76.96)	75.61% (95% CI: 32.67–91.16)	*p* = 0.0064
Second-line, four	1.29 (95% CI: 0.83–2.0)	79.75% (95% CI: 77.38–82.02)	70.31% (95% CI: 62.03–77.97)	99.31% (95% CI: 99.02–99.52)	*p* = 0.252
Only high-quality meta-analyses, three	1.29 (95% CI: 1.02–1.62)	84.14% (95% CI: 79.79–88.06)	66.85% (95% CI: 50.33–81.47)	99.40% (99.10–99.61)	*p* < 0.0001

## Data Availability

No new data were created or analyzed in this study. Data sharing is not applicable to this article.

## Supplementary Materials

The following supporting information can be downloaded at: https://www.mdpi.com/article/10.3390/jpm15100458/s1. File S1. Completed PRISMA-P checklist. File S2: Forest Plot of the Pooled Relative Risk for Tailored vs. Empirical Therapy; Pooled Relative Risk for Tailored vs. Empirical Therapy as First-Line Treatment for *H. pylori* Eradication; Pooled Relative Risk for Tailored vs. Empirical Therapy as Second-Line Treatment for *H. pylori* Eradication; Pooled Relative Risk for Tailored vs. Empirical Therapy Based on High-Quality Meta-Analyses; Publication Bias Assessment.
