# Supplementary material for: Tailored Therapy vs. Empirical Therapy for Helicobacter pylori Eradication: An Umbrella Review of Systematic Reviews and Meta-Analyses
Source: J Pers Med. 2025 Sep 30;15(10):458. doi: 10.3390/jpm15100458 (PMC12565390; doi:10.3390/jpm15100458)
Supplement: Supplementary file 1 [file jpm-15-00458-s001.zip › File S2.pdf]

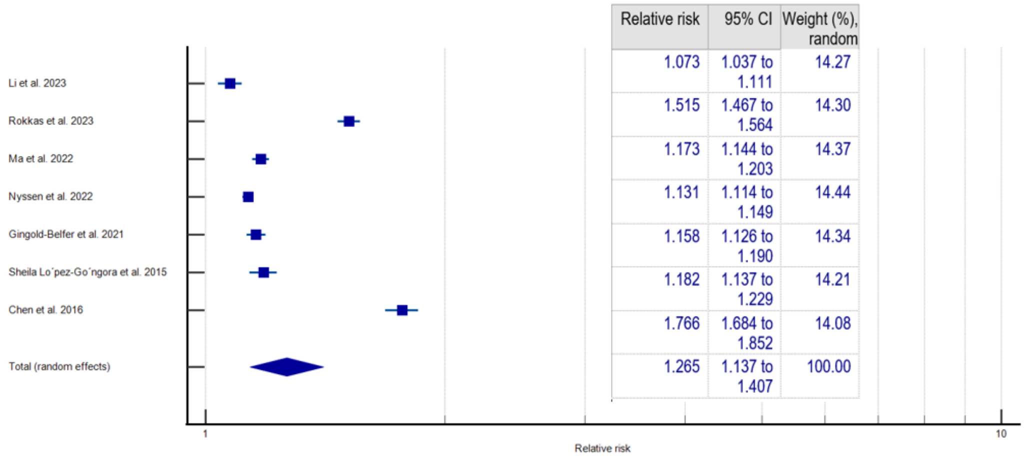

Relative risk	95% CI	Weight (%), random
1.073	1.037 to 1.111	14.27
1.515	1.467 to 1.564	14.30
1.173	1.144 to 1.203	14.37
1.131	1.114 to 1.149	14.44
1.158	1.126 to 1.190	14.34
1.182	1.137 to 1.229	14.21
1.766	1.684 to 1.852	14.08
1.265	1.137 to 1.407	100.00

Forest Plot of the Pooled Relative Risk for Tailored vs. Empirical Therapy

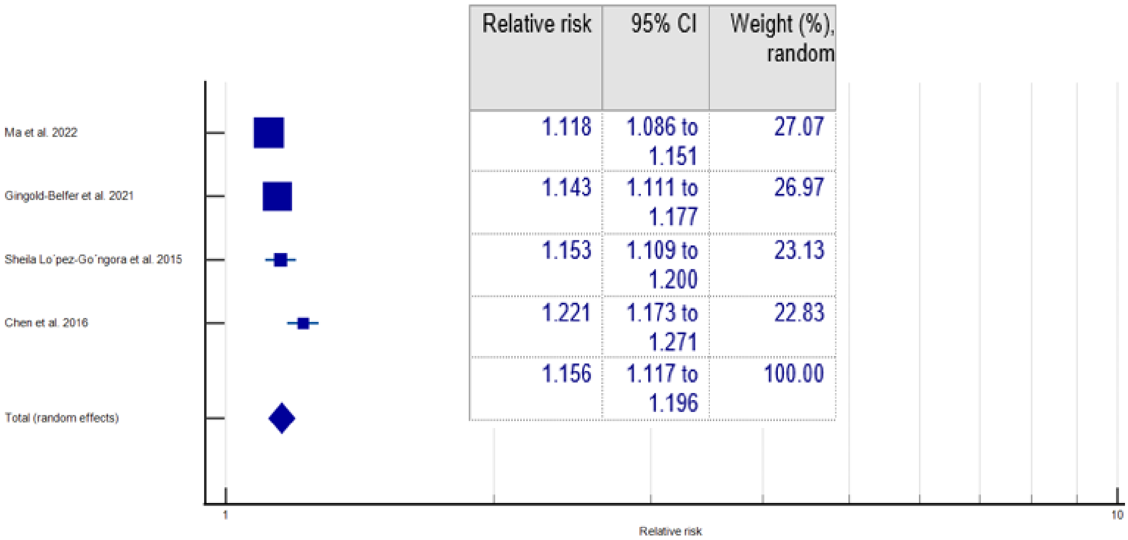

Relative risk	95% CI	Weight (%), random
1.118	1.086 to 1.151	27.07
1.143	1.111 to 1.177	26.97
1.153	1.109 to 1.200	23.13
1.221	1.173 to 1.271	22.83
1.156	1.117 to 1.196	100.00

Pooled Relative Risk for Tailored vs. Empirical Therapy as First-Line Treatment for *H. pylori* Eradication

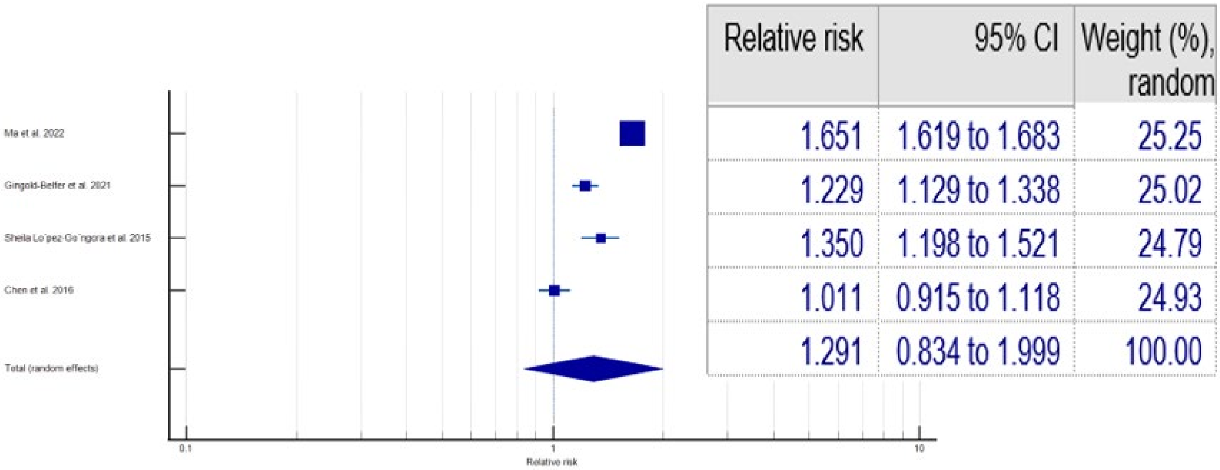

Relative risk	95% CI	Weight (%), random
1.651	1.619 to 1.683	25.25
1.229	1.129 to 1.338	25.02
1.350	1.198 to 1.521	24.79
1.011	0.915 to 1.118	24.93
1.291	0.834 to 1.999	100.00

Pooled Relative Risk for Tailored vs. Empirical Therapy as Second-Line Treatment for *H. pylori* Eradication

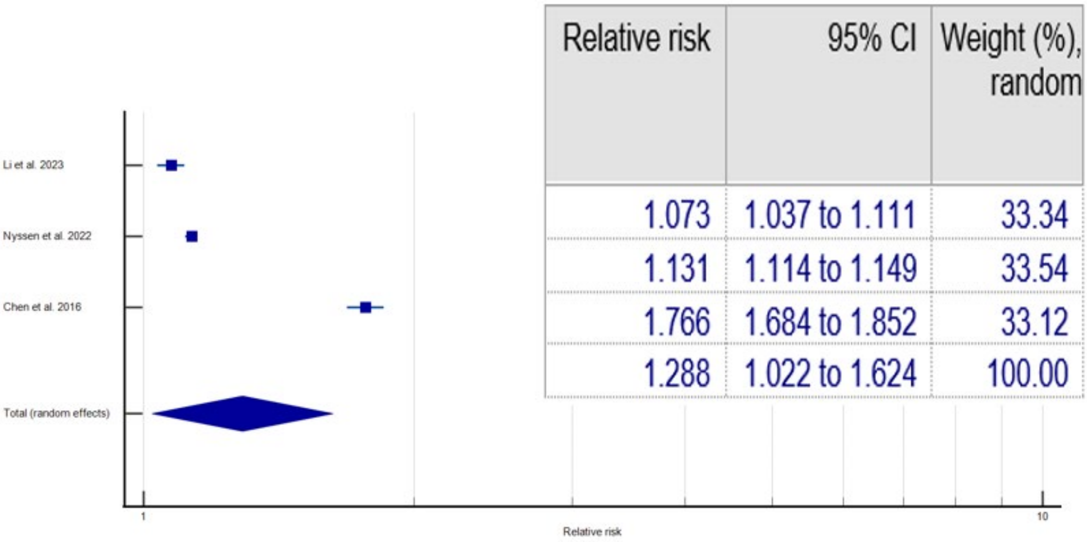

Pooled Relative Risk for Tailored vs. Empirical Therapy Based on High-Quality Meta-Analyses

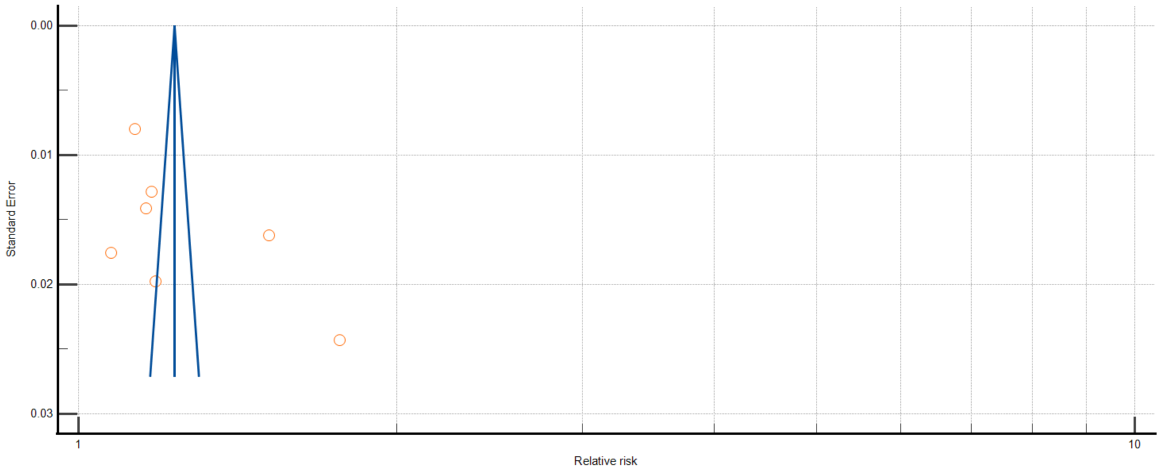

Publication Bias Assessment
